# Volumizing effects of a smooth, highly cohesive, viscous 20-mg/mL hyaluronic acid volumizing filler: prospective European study

**DOI:** 10.1186/1471-5945-9-9

**Published:** 2009-08-27

**Authors:** Klaus Hoffmann

**Affiliations:** 1 Ruhr-University Bochum, St. Josef Hospital, Bochum, Germany

## Abstract

**Background:**

Facial volume loss contributes significantly to facial aging. The 20-mg/mL hyaluronic acid (HA) formulation used in this study is a smooth, highly cohesive, viscous, fully reversible, volumizing filler indicated to restore facial volume. This first prospective study evaluated use in current aesthetic clinical practice.

**Methods:**

A pan-European evaluation conducted under guidelines of the World Association of Opinion and Marketing Research, the trial comprised a baseline visit (visit 1) and a follow-up (visit 2) at 14 ± 7 days posttreatment. Physicians photographed patients at each visit. Each patient was treated with the 20-mg/mL HA volumizing filler as supplied in standard packaging. Procedural details, aesthetic outcomes, safety, and physician and patient ratings of their experience were recorded.

**Results:**

Fifteen physicians and 70 patients (91% female; mean age: 50 years) participated. Mean volume loss at baseline was 3.7 (moderate) on the Facial Volume Loss Scale. Local anesthesia was used in 64.3% of cases. Most injections (85%) were administered with needles rather than cannulas. Of the 208 injections, 59% were in the malar region, primarily above the periosteum. Subcutaneous injections were most common for other sites. The mean total injection volume per patient was 4.6 mL. The mean volume loss score declined significantly (*P *< .001) to 2.1 at visit 2. On the Global Aesthetic Improvement Scale, 88% and 76% of the treatments were rated very much improved or much improved by physicians and patients, respectively. Of the physicians, 95.6% rated this HA filler as very or fairly easy to use. Similarly, 92% of patients were very likely or quite likely to return for treatment; nearly all (98%) would recommend this treatment to friends. Transient (mean duration: 5.5 days) injection-site adverse events (AEs) occurred in 24 patients. Bruising was the most common AE.

**Conclusion:**

The 20-mg/mL smooth, highly cohesive, viscous, volumizing HA filler was effective, well tolerated, and easy to use in current clinical practice. Participants were very likely to recommend this product to colleagues and friends, and patients would be very or quite likely to request this product for future treatments.

## Background

Facial aging is a consequence of multiple, interacting intrinsic and extrinsic factors.[1] Sun exposure, or photoaging, contributes importantly to the intrinsic changes associated with aging. These changes have been described using the Glogau photoaging classification, which focuses on the degree of wrinkling.[2] Another factor in the appearance of facial aging is the lifelong activity of the muscles of facial expression, which produce the dynamic and, ultimately, static facial lines and folds.[3] It has also long been recognized that gravity exerts a toll on the facial structures as tissue loses its elasticity and becomes less able to resist stretching.

Volume deficits resulting from the loss and repositioning of facial fat, as well as from skeletal remodeling, are now increasingly appreciated as a fundamental component of facial aging.[1,4,5] The younger face is characterized by the harmony and balance captured in the classic heart shape or inverted triangle.[1,6] The reversal of this "triangle of beauty" as aging proceeds and as proportions rearrange is considered generally less aesthetically appealing. With these changes, the convexities typical of a youthful appearance tend to flatten and droop.

Recognition of the key role of volume loss in facial aging has resulted in what has been called a paradigm shift in facial rejuvenation, influencing both the types and extent of surgical procedures, as well as the way in which minimally invasive approaches are employed.[7] Facial rejuvenation has moved beyond wrinkle effacement and surgical facelifts to an approach that relaxes muscles and volumizes, shapes, recontours, and retexturizes. In combination with preventive skin care measures, this comprehensive, multimodal approach permits clinicians to develop aesthetic treatment plans tailored to individual patient needs and designed to produce a natural, relaxed appearance.[6,8]

Subdermal fillers can form a cornerstone of the new facial rejuvenation paradigm. The product that was the subject of this study, a 20-mg/mL smooth, highly cohesive, viscous HA volumizing filler, shares many of the properties of its wider family of 24-mg/mL smooth, cohesive HA fillers, but also has unique physical and chemical properties that make it ideally suited for volumizing and contouring. These products are smooth, cohesive HA fillers as a result of the manufacturing process in which they are homogenized rather than sieved to produce the final product.[9] This is in comparison with hard and granular formulations produced by the sieving process. [9,10] The 20-mg/mL smooth, cohesive HA filler was designed to be more viscous and robust with a higher lift capacity than other members of its family. The 20-mg/mL smooth, cohesive HA filler is derived primarily from low-molecular-weight (LMW) HA. Its HA concentration, 20 mg/mL, and its water binding capacity is similar to that of other 24-mg/mL smooth, cohesive products. Crosslinking of LMW HA results in significantly increased viscosity. As a result, the final product has very high viscosity and cohesivity while remaining soft and readily extrudable. It also retains the properties of reversibility, in the event of overcorrection or complications, and resorbability. At the same time, the product has the potential for long-term persistence in tissue.

The 20-mg/mL smooth, highly cohesive, viscous HA volumizing filler has been evaluated retrospectively in patients with age-related volume loss and prospectively in patients with human immunodeficiency virus (HIV)-associated lipoatrophy.[1,11] The results of both of these studies demonstrated that the 20-mg/mL smooth, cohesive HA volumizing filler was highly effective and well tolerated. Aesthetic improvements were also durable, with reported longevities of at least 12 and up to 18 months based on the defined durations of each study.[1,11]

In this article, we report on the first prospective trial designed to assess how the 20-mg/mL smooth, cohesive HA volumizing filler is used in current clinical practice. Specific objectives were to characterize the aesthetic benefit and safety profile of the 20-mg/mL smooth, cohesive HA volumizing filler; to evaluate patient and clinician perspectives on the product; to assess practice preferences, including similarities, and differences among specialties; and to derive practice recommendations.

## Methods

### Study Design

This was a prospective, open-label, nonrandomized study in which a 20-mg/mL smooth, highly cohesive, viscous HA volumizing filler (Juvéderm™ VOLUMA™, Allergan, Pringy, France) was evaluated within its indicated use of restoring facial volume. The study was funded by Allergan, Inc. This Europe-wide evaluation was conducted under the guidelines of the World Association of Opinion and Marketing Research (ESOMAR) to evaluate current usage of the 20-mg/mL smooth, cohesive HA volumizing filler in European countries in which the product is CE-marked or licensed and available. Evaluations took place within standard practice procedures without the inclusion of any additional monitoring or diagnostic procedures. The conduct of the trial complied with the provisions of the Helsinki Declaration for studies in humans; however, it was not a clinical trial as such. As a market research evaluation of a CE-marked device within intended use, regulatory approval is not required, and therefore, ethics approval was not sought.

### Patients

Men and women of at least 30 years of age who requested treatment for facial volume loss and who were able to provide informed consent were eligible for participation. They were required to have a facial volume loss score of at least 2 on the 5-point Facial Volume Loss Scale.[12] Prospective patients were excluded if they were participants in any ongoing clinical trial or had any conditions contraindicating the use of the 20-mg/mL smooth, cohesive HA volumizing filler, such as a tendency to develop hypertrophic scars, known hypersensitivity to HA, or pregnancy or lactation.[13] The presence of inflamed or infected skin (eg, acne, herpes) in the planned treatment areas precluded participation. Also excluded were patients who had received any previous treatment with a permanent or semipermanent filler or implant within the preceding 12 months.

### Study Procedures

**Figure 1:**
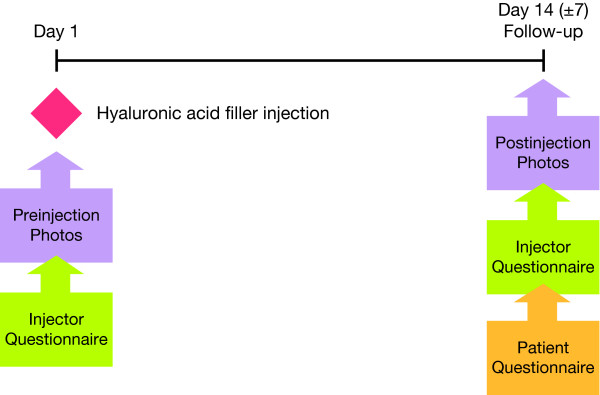
**Study flow and treatment**.

The study comprised 2 visits. Visit 1 was the injection day, which took place on day 1. Visit 2 was the follow-up, which took place at day 14 (± 7 days) (Figure 1). Case assessment forms were completed by each investigator at each visit and served as the basis for data collection. This included details on the clinical practice and physician specialty, patient age and sex, and all outcome variables. Pre- and posttreatment digital photography of frontal, lateral, and oblique views were taken at both visits. Patients completed a questionnaire at the second visit to rate their perceptions of their experience.

Investigators injected the 20-mg/mL smooth, cohesive HA volumizing filler with either a cannula or needle, according to their usual practices. They determined the volume to be injected based on patient assessment using the Facial Volume Loss Scale (1 = mild, 2 = intermediate between mild and moderate, 3 = moderate, 4 = intermediate between moderate and severe, 5 = severe) and their aesthetic judgment. Touch-ups could be performed at visit 2 based on the judgment of the investigator. The use of anesthetic also depended upon the preferences and usual practice of the treating physician.

### Data Collection and Study Endpoints

Investigators recorded each patient's age, sex, and baseline volume loss according to the Facial Volume Loss Scale. At the time of treatment, investigators recorded the type(s) of anesthesia, the type and size of the injection device (needle or cannula), the injection sites and volume per site, the total volume, and the injection plane and pattern. They also assessed the ease of injection, sculpting, and shaping with the 20-mg/mL smooth, cohesive HA volumizing filler, and recorded their posttreatment instructions for patients.

Effectiveness was assessed at visit 2 and was based on investigator-assessed changes from baseline on the Facial Volume Loss Scale and on investigator and patient ratings on the Global Aesthetic Improvement Scale (GAIS), which includes 5 categories ranging from very much improved to worse.[14] The need for touch-ups was evaluated. This included the reason, the site, the injection technique and plane, and the volume. Safety was monitored by recording the occurrence of any adverse event (AE) that followed treatment, including its site, severity, and duration. Severity was determined by the usual clinical criteria of each individual investigator. At this visit, investigators also noted their willingness to recommend the 20-mg/mL smooth, cohesive HA volumizing filler to their colleagues.

At visit 2, patients completed their questionnaire, recording their satisfaction with the overall cosmetic effect, their likelihood of returning for additional treatments with the 20-mg/mL smooth, cohesive HA volumizing filler, and their willingness to recommend it to their friends.

### Data Analyses

The data were summarized with descriptive statistics (eg, percentages, means, ranges). The differences between pretreatment and posttreatment mean Facial Volume Loss Scale scores were analyzed using a t-test.

## Results

### Study Participants

Fifteen physicians (from France, Germany, Italy, Spain, the United Kingdom, Benelux countries, and Russia [licensed as Voluma Corneal^® ^in Russia]) participated in the study. Of these, 4 were dermatologists, 6 plastic surgeons, and 5 aesthetic practitioners. A total of 70 patients were recruited. The majority were female with a mean age of 50 years (Table 1). Based on the Facial Volume Loss Scale mean score (3.7), patients on average had a somewhat greater than moderate loss of facial volume. Mean ratings by specialty were 3.7, 4.0, and 3.2 for dermatologists, plastic surgeons, and aesthetic practitioners, respectively.

**Table 1:** Patient Baseline and Demographic Data (N = 70)

Characteristic	Value
Sex	**91% female**

Mean age	**50**

Ascher Scale (mean score)	**3.7**

Ascher Scale (score distributions)*	**Grade 2: 44%** **Grade 3: 34%** **Grade 4: 14%** **Grade 5: 3%**

*Total does not equal 100 because 3 patients did not meet the enrollment criterion of a volume loss score of at least 2 at baseline.

### Treatment and Practice Characteristics

The type of anesthesia used was recorded for 56 of the 70 patients. Across all physicians, local anesthesia was used in the majority (64.3%) of the 56 patients (Table 2). Some differences among specialties were apparent. For example, topical anesthetics were used for 44% of treatments by dermatologists and for 27% by plastic surgeons. In contrast, aesthetic practitioners used local anesthesia for 95% of their treatments.

**Table 2:** Type of Anesthesia

Anesthesia (n = 56)			
Local	Topical	Both	Regional or Block

**64.3%**	**19.6%**	**1.8%**	**14.3%**

Needles were used to deliver product for 85% of all injections (Table 3). The median needle gauge was 21 (range: 21–27 gauge).(Excel spreadsheet, Column L [Q5CA]) The median cannula size was 18 gauge (range: 11.6–19 gauge) (Excel spreadsheet column M [Q5DA])

**Table 3:** Type of Injection Device

Device 178 Mentions	
Needle	Cannula

**85%**	**15%**

The most frequent treatment site for each side of the face was the malar area, accounting for 59% of the 208 total injections (Table 4). The mean injection volume in the malar region was 1.9 mL/side. The next most commonly injected site (21% of procedures) was the nasolabial fold area with a mean injection volume of 1.45 mL/side. Across all patients and all areas, the mean total volume per patient was 4.6 mL. Dermatologists and plastic surgeons injected greater total volumes per patient than did aesthetic practitioners. The mean total volume injected per patient by dermatologists was 5.5 mL, by plastic surgeons was 5.4 mL, and by aesthetic practitioners was 3.0 mL.

**Table 4:** Injection Sites and Volumes*

Injection Sites (Mean Volume/Side) 208 Sites Recorded				
Malar	Nasolabial Folds	Chin	Temporal	Other

**59%** **(1.9 mL)**	**21%** **(1.45 mL)**	**9%** **(0.5 mL)**	**7%** **(0.7 mL)**	**5%** **(1.75 mL)**

*Total exceeds 100% because of rounding.

All injections were in the deep dermis or above the periosteum as recommended in the product instructions for use.[13] In the malar area, the majority of injections were subcutaneous (39%) or above the periosteum (40%). In other areas, subcutaneous injections predominated. Linear threading was the most common injection technique for the malar area (36%), chin (59%), and nasolabial folds (51%). Fanning was the most common technique used for the temporal area (58%) and other areas (57%.) Some differences among specialties were noted. Dermatologists used cross-hatching 47% of the time, linear threading 28% of the time, and a combination of techniques 40% of the time. Plastic surgeons used linear threading 37% of the time, followed by fanning (24%), cross-hatching (16%), and combinations (18%). Aesthetic practitioners used primarily cross-hatching (48%), then fanning (22%), and combinations (16%).

### Aesthetic Outcomes

#### Effectiveness

**Figure 2:**
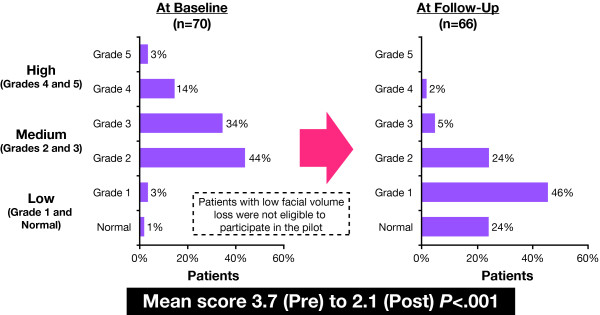
**Volume loss ratings on the Ascher Scale**[12]: **changes from baseline to follow-up**.

The mean scores on the Facial Volume Loss Scale declined significantly (*P *< .001) from 3.7 at visit 1 to 2.1 at Visit 2. The change in score distributions demonstrates that 78% of patients had been rated as a having a medium degree of volume loss pretreatment compared with 29% posttreatment (Figure 2). At visit 2, 70% of patients were rated as having a low degree of volume loss.

**Figure 3:**
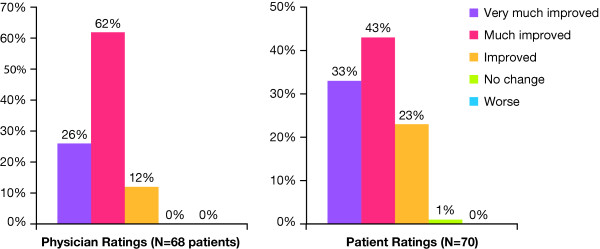
**Physician and patient ratings on the Global Aesthetic Improvement Scale**.

**Figure 4:**
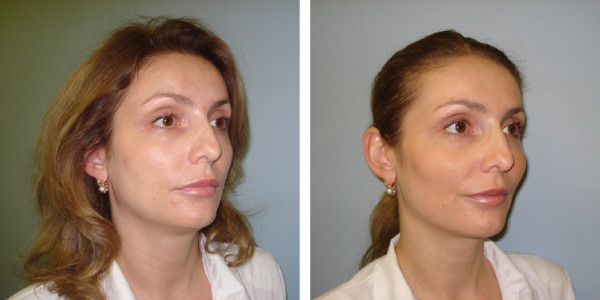
**Typical patient treated with the 20-mg/mL smooth, cohesive HA volumizing filler**. This 37-year-old patient with volume loss of grade 3 on Ascher Scale was injected in each malar region of her face with 2 mL of the 20-mg/mL smooth, cohesive HA volumizing filler. The injections were made above the periosteum using the linear threading technique. Written consent for publication was obtained from the patient.

Investigators rated improvement on the GAIS for 68 patients; 88% were rated as very much or much improved, and the remaining patients (12%) were rated as improved (Figure 3). Of the 70 patients, 76% rated themselves as very much or much improved (Figure 3). Only one patient noted no change in appearance. Thus 99% of the patients rated themselves as improved. Figure 4 illustrates changes in appearance of a typical patient.

#### Touch-Ups

Nine patients (13%) were deemed to require touch-ups. The most common reason was the need for additional volume. Two touch-ups were needed to correct an uneven result. For the right side, 8 patients were treated with an additional mean volume of 0.8 mL. Five patients required an additional mean volume of 0.7 mL on the left side. The total mean additional volume added at visit 2 was 1.5 mL.

#### Posttreatment recommendations

Specific posttreatment recommendations varied considerably across participating physicians but were generally consistent with typical practice for any facial filler. The more commonly mentioned recommendations to patients were to avoid massaging the area for at least 24 hours, sauna treatments, vigorous sports, sleeping face-down, and anticoagulant medications such as aspirin. Patients were also cautioned to expect the possibility of bruising and swelling.

### Safety

The majority (66%) of the 70 patients experienced no AEs. A total of 24 patients experienced injection-site reactions (Table 5). The mean duration of these events was 5.5 days (range: 2–15 days). Seven of the AEs were rated as severe (bruising and swelling, n = 2; pain and swelling, n = 2, pain, n = 1; infection and swelling, n = 1, pain and bruising, n = 1).

**Table 5:** Adverse Events

Event	Number of Patients (n = 24)
Bruising	7

Pain and bruising	4

Swelling	3

Bruising and swelling	3

Pain and swelling	2

Pain	2

Bruising and bleeding	1

Infection and swelling	1

Not specified	1

For the patient who experienced posttreatment infection, previous aesthetic treatments included onabotulinumtoxintypeA (BOTOX^®^), synthetic polyacrylamide gel polymer filler (Bio-Alcamid™; Rofil), and lip augmentation with synthetic polyacrylamide gel polymer filler (Beautical 2^®^; Rofil). Prior polyacrylamide treatments were not disclosed to the treating physician. Four months after injection with 2 mL of the 20-mg/mL smooth, highly cohesive, viscous HA volumizing filler into the malar area and chin; a biofilm and abscess developed. The event was determined to be unrelated to treatment with the 20-mg/mL smooth, highly cohesive, viscous HA volumizing filler. The patient was treated with amoxicillin/clavulanic potassium (Augmentin^®^) 3 times daily for 10 days, and with ibuprofen 600 mg, 3 times daily for 7 days. The site was evacuated and cleansed, and the symptoms fully resolved without further sequelae.

### Physician Experience and Perspectives on Treatment

**Figure 5:**
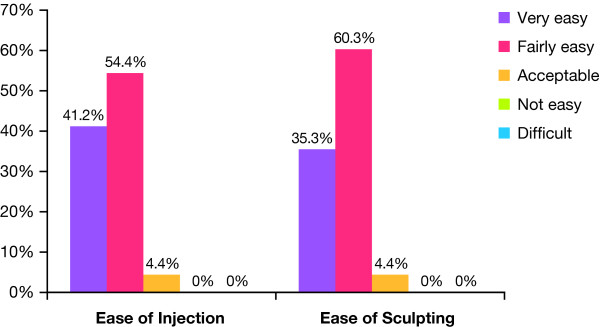
**Physician ratings: ease of use and sculpting**.

When asked to rate ease of use, approximately 95.6% of investigators rated the 20-mg/mL smooth, cohesive HA volumizing filler as very or fairly easy to inject, regardless of injection technique or plane (Figure 5). Similarly, 96% of investigators rated the product as very or fairly easy to sculpt or shape in the majority of patients. Physicians were asked to note whether they would recommend the 20-mg/mL smooth, cohesive HA volumizing filler to colleagues after each treatment they completed. Data were available for 67 treatment sessions, of which 98% were positive.

### Patient Experience and Perspectives

**Figure 6:**
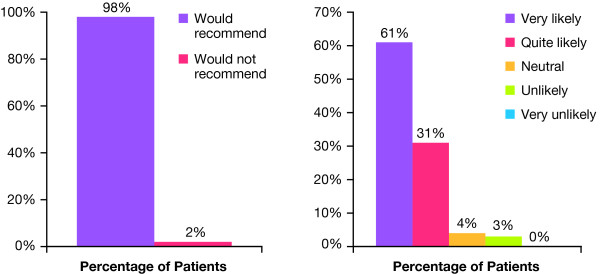
**Patient ratings**. Percentage of patients who would recommend the 20-mg/mL smooth, cohesive HA volumizing filler to friends. Likelihood of returning for additional treatment with the 20-mg/mL smooth, cohesive HA volumizing filler.

Patient experience with the 20-mg/mL smooth, cohesive HA volumizing filler was highly positive, with 98% of patients reporting that they would recommend the treatment to their friends (Figure 6). Approximately 92% of the patients noted that they would be quite likely or very likely to return for treatment (Figure 6).

## Discussion

To our knowledge, this was the first prospective evaluation on the use of a 20-mg/mL smooth, highly cohesive, viscous HA volumizing filler in current aesthetic clinical practice. The study was designed to characterize treatment techniques, assess effectiveness and safety, and evaluate physician and patient perspectives on the experience. Patients with facial volume loss of at least 2 on the Facial Volume Loss Scale were eligible. A total of 70 patients and 15 investigators (dermatologists, plastic surgeons, and aesthetic practitioners) participated.

Most injections were performed under local anesthesia and needles were used in the majority of treatments, although both needles and cannulas will be available in the supplied standard product package. The most common treatment area was the malar region with a mean total injection volume per patient of 4.6 mL. Regardless of technique, treatment area, or specialty, the vast majority of physicians found the 20-mg/mL smooth, cohesive HA volumizing filler to be easy to inject, sculpt, and mold.

Treatment with the 20-mg/mL smooth, highly cohesive, viscous HA volumizing filler resulted in statistically significant improvements in facial volume. Indeed, 70% of patients were rated as having a low degree of volume loss posttreatment, a substantial change from pretreatment. The vast majority of patients and physicians rated the changes in appearance as much or very improved, and the majority of patients did not experience any AEs. Based on patient ratings, including their high likelihood of returning for treatment and of recommending it for friends, the outcomes provided a high degree of patient satisfaction, one of the most important outcomes in aesthetic medicine. The results from this study are consistent with those of the first retrospective analysis of the 20-mg/mL smooth, highly cohesive, viscous HA volumizing filler (Juvéderm™ VOLUMA™) in a case series of 102 patients with age-related facial volume loss.[1] This retrospective study, however, differed from the current study in the volume loss scale that was used, and that patients were evaluated at 30 days after treatment to allow all potential treatment site responses to fully subside. Data from other published studies also reveal that the 20-mg/mL smooth, highly cohesive, viscous HA volumizing filler provides a durable response of at least 12 to 18 months when used for restoring facial volume lost through aging or resulting from HIV-associated lipoatrophy.[1,11] It is very important to note that there were no instances of migration or mobility of the product, nor any irregularities in appearance in the injection area. In contrast, clinical experience with the 20-mg/mL 1,000 particle, granular consistency HA filler (Restylane^® ^SubQ) has revealed migration from the injection area, which is speculated to be the result of its low cohesivity and nonhomogenous formulation. Together, the properties of the 20-mg/mL smooth, highly cohesive, viscous HA volumizing filler render it highly suitable for use in the malar and chin areas. Note that in this study, very experienced investigators used the product for deep injections into the nasolabial folds, but it is not indicated or recommended for routine use in either this area or in the lips, as they require more superficial treatment. Other products, like the 24-mg/mL smooth, cohesive HA filler and other HAs are more suitable for use in these areas. Caution is also warranted when injecting additional fillers in patients who have previously been treated with synthetic polyacrylamide gel polymer fillers.

Volumizing procedures require somewhat greater skill than more superficial dermal filling procedures; specifically, that injections should be placed into the deep dermis or above the periosteum, and should be placed under the orbicularis oculi, but strictly above the deep zygomaticus. The temporal branch of the facial nerve should be avoided. Likewise, it is important to educate patients about the differences between volumizing procedures and dermal filling. Patients should be advised that swelling may occur and is not unusual. Nevertheless, 66% of the patients experienced no AEs. Observed AEs were consistent with those expected when volumizing the face and were primarily local (bruising and pain) and self-limited with a mean duration of 5.5 days.

The variations we observed in techniques, including use and type of anesthesia, needle versus cannula, planes of injection, and in injection techniques (eg, linear threading, cross-hatching, fanning) suggest that the product can be used successfully according to the individual physician's or surgeon's preference. There are advantages and limitations to both cannulas and needles, both of which are supplied with the product. For example, needle use may be easier than the cannula and may offer greater precision. Tissue trauma, however, and the risk of hematoma may be greater with multiple needle injection sites. It should be noted that these risks can be reduced by using a needle and administering only 1 or 2 injections.[1] The cannula technique may result in less bleeding, but it looks more aggressive. This must be balanced against the potential for a lower degree of precision. In this study, very few patients required touch-ups when evaluated approximately 2 weeks posttreatment. In actual practice, we recommend evaluating for touch-ups at 1 month posttreatment to be sure that all edema, however minimal, have completely resolved and HA water absorption is complete.

## Conclusion

This 20-mg/mL smooth, highly cohesive, viscous, fully reversible, resorbable, HA volumizing filler is indicated for restoring facial volume loss.[13] In practice this may result from aging, congenital anatomic defects, or HIV-associated lipoatrophy.[1,11] As shown in this study, the physical and chemical properties of the 20-mg/mL smooth, cohesive, HA volumizing filler make it robust and ideal for volumizing, yet still easy to inject, mold, and sculpt. It should be emphasized that volumizing is not a procedure to be undertaken without thorough knowledge of facial anatomy and physiology nor without proper training because it differs from the more superficial procedures with fillers. The results of this prospective evaluation of actual clinical practice demonstrate that the 20-mg/mL smooth, cohesive, HA volumizing filler provides a very effective, well-tolerated approach to volumizing an aging face. Participants did not report any occurrences of product migration, which differs from reported clinical experience with the 20-mg/mL 1,000 particle, granular consistency HA filler. The 20-mg/mL smooth, highly cohesive, viscous HA volumizing filler provided a high degree of satisfaction to patients.

## Competing interests

Klaus Hoffmann has received payments as a consultant for Allergan, Inc. Allergan, Inc. manufactures the 20-mg/mL smooth, highly cohesive, viscous, fully reversible, resorbable, HA volumizing filler (Juvéderm™ VOLUMA™). Allergan provided samples of Juvéderm™ VOLUMA™ (enough samples for the evaluation only) to the investigators. Additional funding or honoraria was not provided to the investigators.

## Consent

Written informed consent was obtained from the patient for publication of this report and accompanying images. A copy of the written consent is available for review by the Editor-in-Chief of this journal.

## Authors' contributions

The Juvéderm Voluma Study Investigators Group agreed to authorship by KH who made substantial contributions to the conception and study design, acquisition of data, and analysis and interpretation of data. The author was involved in drafting and revising the manuscript critically and has given final approval of the version to be published.

## Author information

Klaus Hoffmann is a dermatologist at the St. Josef-Hospital Bochum, Ruhr-University Bochum, Bochum, Germany.

## Pre-publication history

The pre-publication history for this paper can be accessed here:

http://www.biomedcentral.com/1471-5945/9/9/prepub
